# Human Milk Cells Contain Numerous miRNAs that May Change with Milk Removal and Regulate Multiple Physiological Processes

**DOI:** 10.3390/ijms17060956

**Published:** 2016-06-17

**Authors:** Mohammed Alsaweed, Ching Tat Lai, Peter E. Hartmann, Donna T. Geddes, Foteini Kakulas

**Affiliations:** 1 School of Chemistry and Biochemistry, The University of Western Australia, Crawley, WA 6009, Australia; mohammed.alsaweed@research.uwa.edu.au (M.A.); ching-tat.lai@uwa.edu.au (C.T.L.); peter.hartmann@uwa.edu.au (P.E.H.); donna.geddes@uwa.edu.au (D.T.G.); 2 College of Applied Medical Sciences, Majmaah University, Almajmaah, Riyadh 11952, Saudi Arabia

**Keywords:** human milk, breastmilk, miRNA profiling, milk cells, next generation sequencing, milk removal, breastfeeding

## Abstract

Human milk (HM) is a complex biofluid conferring nutritional, protective and developmental components for optimal infant growth. Amongst these are maternal cells, which change in response to feeding and were recently shown to be a rich source of miRNAs. We used next generation sequencing to characterize the cellular miRNA profile of HM collected before and after feeding. HM cells conserved higher miRNA content than the lipid and skim HM fractions or other body fluids, in accordance with previous studies. In total, 1467 known mature and 1996 novel miRNAs were identified, with 89 high-confidence novel miRNAs. HM cell content was higher post-feeding (*p* < 0.05), and was positively associated with total miRNA content (*p* = 0.014) and species number (*p* < 0.001). This coincided with upregulation of 29 known and 2 novel miRNAs, and downregulation of 4 known and 1 novel miRNAs post-feeding, but no statistically significant change in expression was found for the remaining miRNAs. These findings suggest that feeding may influence the miRNA content of HM cells. The most highly and differentially expressed miRNAs were key regulators of milk components, with potential diagnostic value in lactation performance. They are also involved in the control of body fluid balance, thirst, appetite, immune response, and development, implicating their functional significance for the infant.

## 1. Introduction

Human milk (HM) is a complex system of nutritional and bioactive components that together offer the essential building blocks for the optimal growth, development and protection of the infant [1,2]. The latest research has shifted focus from the nutritional components of HM, which have been well studied, to its bioactive elements, including maternal cells and the molecules they synthesize and secrete, such as microRNAs (miRNAs) [3,4,5]. The maternal cells of HM are primarily of mammary epithelial origin when the mother and infant are healthy, but they are dominated by immune cells originating from the maternal circulation in the first days postpartum (colostrum) and during periods of infection of either the mother or the infant [4,6,7]. miRNAs are small non-coding RNA molecules (~22 nucleotides long) [8,9,10,11] that are present in all three fractions of HM (cells, lipids and skim milk) [8,9,11,12], and have been shown to originate mainly from mammary epithelial cells [12]. They are potent regulators of gene expression at the post-transcriptional level [13] and are involved in several biological processes including apoptosis, cell differentiation, development and growth of various tissues and organs [14]. miRNAs are abundant in HM compared to other body fluids, such as plasma and peripheral blood mononucleated cells [8,9,11,12,15], and are likely to be transferred to the infant via the exosomal and cellular HM components [3,16,17].

The roles of HM miRNAs in the infant have not yet been explored. However, most recently, food-derived miRNAs, including those derived from commercially available bovine milk, were demonstrated to integrate into human and animal cells *in vitro*, and also survive the gastrointestinal tract *in vivo*, being transferred to the systemic circulation and various organs, where they exert gene regulatory functions at the cellular level [18,19,20,21,22,23,24,25]. Some of these miRNAs were further shown to have therapeutic effects *in vivo*, including amelioration of experimental rheumatoid arthritis in a mouse model [22]. In addition, due to the lack of supporting evidence for transfer of certain plant-derived miRNAs into mammalian cells [19,26,27], it has been proposed that the packaging of miRNAs in transporting ‘vehicles’, including exosomes and somatic cells, which is typical of milk miRNAs, plays an essential role in their survival and integration in mammals [3]. This, together with the high stability of miRNAs under harsh conditions [8,10], and given that the infant’s stomach is less acidic and the gut more permeable than adults [28], have provided strong support for the survival, absorption and integration of HM miRNAs in the infant, and their potential functional significance in early growth, protection, and development. It is, therefore, important to characterize the miRNA content of HM and examine factors that may influence it.

Milk miRNAs have mostly been studied in the skim milk fraction [8,9,29,30,31], with recent studies in milk lipids [11,15] and cells [12] showing that skim milk is not as rich in miRNA content and composition as the other two milk fractions, and that milk cells conserve more miRNA species than milk lipids. Indeed, this is also the case for blood plasma compared to blood cells [12]. Yet, studies on the miRNA content of milk cells are scarce [12], with no study to our knowledge examining the milk cellular miRNA profile using deep sequencing for discovery of novel miRNAs. In 2013, Munch *et al.* used next generation sequencing in the HM lipid fraction and detected 21 novel miRNAs [11]. It is likely that HM cells, being richer in miRNAs, conserve more novel miRNAs than HM lipids, which may be influenced by milk removal. Cell content is known to increase post-feeding in HM [32], similar to fat content [32,33], suggesting that changes in the milk miRNA content and composition may also occur with feeding. To further examine the effects of milk removal and characterize the miRNA profile of the cells of HM, we purified and quantified miRNAs from the cell fraction of HM collected before and after feeding from exclusively breastfeeding mothers in month 2 of lactation, and performed deep sequencing using the Illumina (Solexa) platform.

## 2. Results

### 2.1. Human Milk Cells Contain Numerous Known and Novel miRNAs

**Figure 1:**
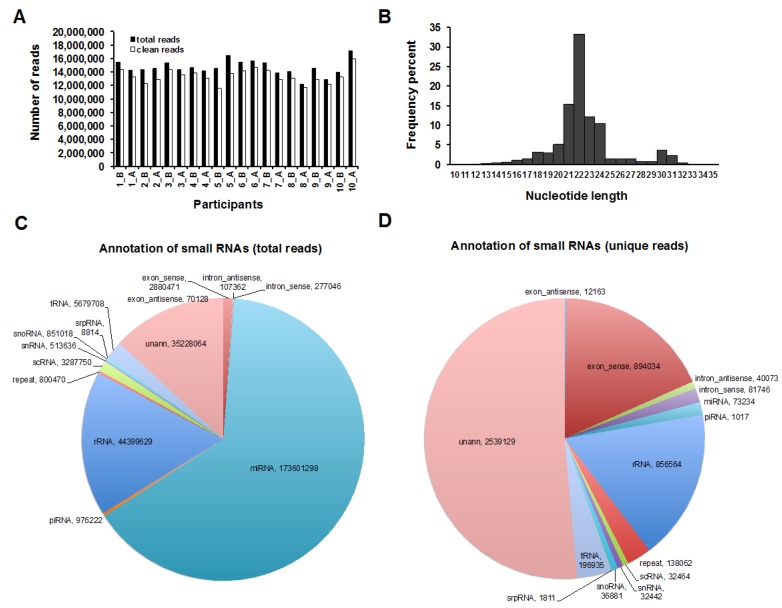
Filtering and annotation of the small RNA reads. (**A**) The total number of small RNA raw reads including miRNAs, and the total number of clean reads that were generated after filter analysis; (**B**) Percentage of the nucleotide length distribution of clean small RNAs after filter analysis; (**C**) Total clean reads were annotated to one or more RNA categories (*i.e.* rRNA, tRNA, scRNA *etc.*); (**D**) Small RNAs were mapped to only one RNA category using the following priority rule: rRNAetc (in which Genbank > Rfam) > known miRNA > piRNA > repeat > exon > intron3. rRNAs were used as a marker of sample quality, with the criterion of high quality when <40% in each sample.

Species and expression levels of known and novel miRNAs were examined using Illumina HiSeq 2000 (Illumina, San Diego, CA, USA) in pre- and post-feed HM samples collected from a subgroup of 10 mother/infant dyads. By using two sequencing lanes, 293,932,547 reads were generated from all samples, in which 268,681,616 (91.40%) were determined as clean reads of small RNAs (Figure 1A) after filtering analysis. Length distribution analysis was done on the clean reads where a highly percentage (61.42%) of the reads were distributed between 21–23 nucleotides in length, which is considered as the ideal transcript length for miRNAs (Figure 1B). The total and unique clean reads were matched to Genebank and RFam to identify and remove tRNA, rRNA, snRNA, snoRNA, and repeat small RNAs (Figure 1C,D). Thereafter, 208,829,362 reads were retained for miRNA analysis, consisting of 2,612,363 unique sequences. Retained small RNA reads were aligned to human genome using SOAP to analyze expression and distribution of these small RNAs on the genome (Figure S1). All retained reads (208,829,362) were matched to miRBase 21.0 (http://www.mirbase.org/) using BLAST to identify known miRNAs. Matched miRNA sequences were analyzed to determine the base bias on the first position with certain length and on each position (Figure S2A,B). Unannotated reads (unmapped reads) to the human genome that did not map to any other RNA classes, including miRNAs that were identified as known in miRBase, were used for novel miRNA prediction analysis (Figure 1C,D). To predict hairpin structure of miRNA precursors for unannotated mature miRNA reads, the mireap software (http://sourceforge.net/projects/mireap/) was used to explore the secondary structure, the Dicer cleavage site and the minimum free energy of the unannotated small RNA reads.

With no mismatched allowed between the profiled sequences and miRBase, 1467 known miRNAs were identified, consisting of 174,186,534 reads (Table 1; Table S1). Of these, 1287 known miRNAs (total reads = 85,573,127) were detected in pre-feed HM, whilst 1308 known miRNAs (total reads = 88,613,407) in post-feed HM (Table 2). Moreover, a total of 1996 miRNA species was discovered as novel miRNAs in all samples (Table 1 and Table S2). Of these, 961 novel miRNAs (14,202 reads) were identified in pre-feed HM, whilst 1215 novel miRNAs (17,121 reads) in post-feed HM (Table 2). To narrow down the novel miRNA species number to those of high confidence, we selected those predicted novel miRNAs with ≥20 reads and which were identified in ≥4 out of 20 pre- and post-feed milk samples. Using this criterion, 89 novel miRNAs were identified in all samples tested, with a total of 15,337 reads (Table S3).

**Figure 2:**
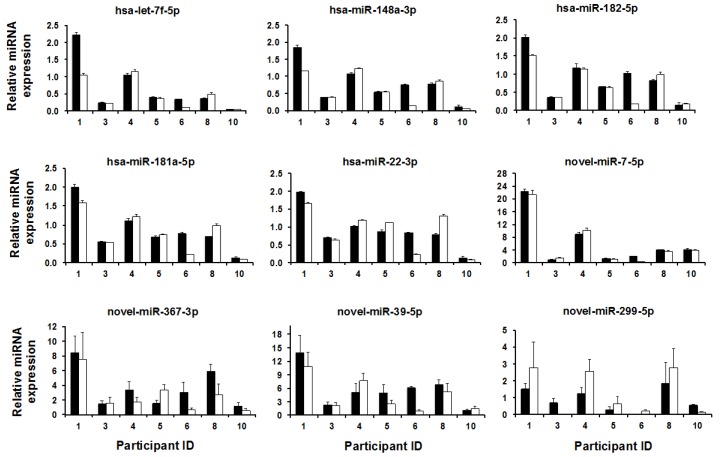
Bar plots showing the expression pattern of the top 5 most highly expressed known miRNAs, and the top 4 most highly expressed novel miRNAs using qPCR. *Y* axes indicate the expression of each miRNA relative to the endogenous control used (RNU48), whilst *X* axes indicate Participant ID. A subgroup of 7 mothers was used for qPCR validation. Linear mixed effects modeling showed no significant differences (*p* > 0.05) in expression of these known and novel miRNAs between pre- and post-feed milk samples for each mother. novel-miR-299-5p was not detected in sample ID 6 (pre-feed milk) and in sample ID 3 (post-feed milk).

The hairpin structure of novel miRNA precursors were predicted for all identified novel mature miRNAs, and the structures of the high confidence novel miRNAs are listed in Table S4. qPCR was used to validate the presence of the top 4 most highly expressed novel miRNAs across samples. Except novel-miR-299-5p, which was not expressed in 2 samples (pre-feed milk sample 6, and post-feed milk sample 3), all other 3 novel miRNAs tested were expressed at high levels in all samples (Figure 2).

**Figure 3:**
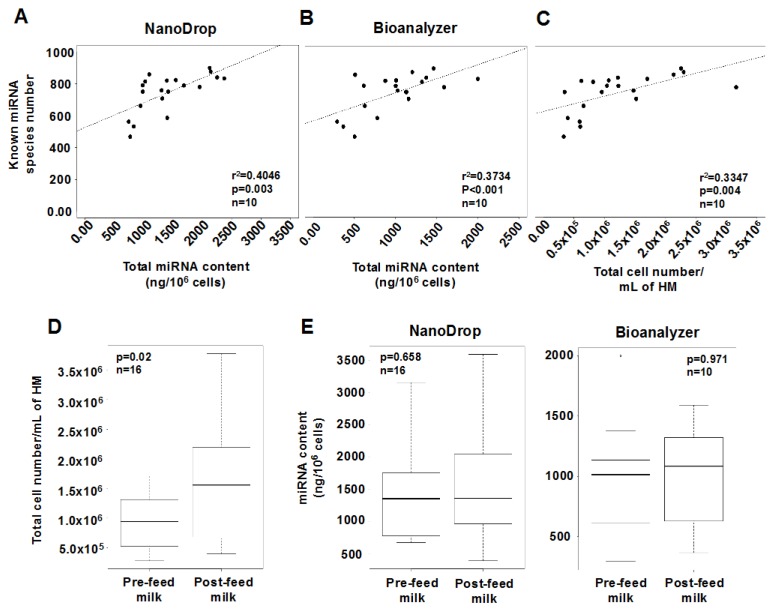

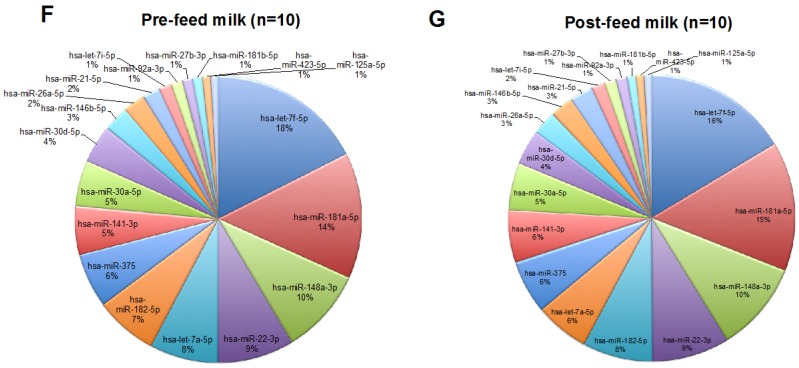
Comparison of HM cell content, total miRNA content, and profiled miRNA species between pre- and post-feed milk. (**A**,**B**) A positive association was found between the total miRNA content and the number of known miRNAs, using either NanoDrop or Bioanalyzer; (**C**) Positive relationship between total cell content per mL of HM and the number of known miRNA species; (**D**,**E**) Comparisons of total cell content per mL of HM and miRNA content (ng/10^6^) between pre- and post-feed milk. The top 19 most highly expressed miRNA species were identical in pre-feed milk (**F**) and post-feed milk (**G**), and accounted for 86.2% of the total miRNAs in all samples (*n* = 20).

Higher cell content in either pre- or post-feed milk was associated with greater miRNA content measured using the Bioanalyzer (*p* = 0.014, *n* = 20 samples). In turn, greater miRNA content was associated with higher number of known and total (known + novel) miRNA species (*p* = 0.003 and *p* < 0.001, respectively, *n* = 20 samples) (Figure 3A,B); however the same was not observed for novel miRNAs (*p* = 0.679, *n* = 20 samples). Similarly, greater cell content was associated with more known and total (known + novel) miRNAs (*p* = 0.004 and *p* < 0.001, respectively, *n* = 20 samples), but not novel miRNAs (*p* = 0.644, *n* = 20 samples) (Figure 3C). The volume taken by the infant was not related to known (*p* = 0.50, *n* = 10) or novel (*p* = 0.90, *n* = 10) miRNA species number. Interestingly, an inverse relationship was found between the number of identified known and novel miRNAs, where samples with high number of novel miRNA species had low number of known miRNA species, and vice versa (*p* = 0.02, *n* = 10).

### 2.2. Effects of Feeding on the miRNA Content of Human Milk Cells

Overall, the total number of cells per mL of HM was higher post-feeding compared to pre-feeding (*p* = 0.028, *n* = 16) (Figure 3D; Table 3), and this increase was not related to the milk volume taken by the infant (*p* = 0.207, *n* = 16). It is of note that in the smaller subgroup of *n* = 10 mothers, the change in cell content post-feeding did not reach significance (*p* = 0.668, *n* = 10). HM fat content was significantly higher in post-feed milk (*p* < 0.001, *n* = 16) than pre-feed milk, and this change was related to the volume taken by the infant (*p* = 0.025, *n* = 16). Further, a positive association was found between fat content and cell content of HM (*p* = 0.006, *n* = 16) (Figure S3A,B). Within a dyad, the total miRNA content per 10^6^ cells did not differ between pre- and post-feed milk (*p* = 0.658 with NanoDrop, *n* = 16; *p* = 0.971 with Bioanalyzer, *n* = 10) (Figure 3E; Table 3), even after accounting for the milk volume taken by the infant. In 7 out of 10 participants, 9-167 additional total (known + novel) miRNA species (76 ± 60 mean ± standard deviation) were detected in post-feed milk. In 3 out of 10 participants, the number of miRNA species was similar between pre- and post-feed milk. Interestingly, in those participants, the differences in cell content pre- and post-feeding were also minimal compared to the other participants. In these 10 mothers, no statistically significant difference in the known or novel or total (known + novel) miRNA species number was found between pre- and post-feed milk within a dyad (*p* = 0.756, *p* = 0.509, and *p* = 0.412, respectively, *n* = 10), and this was not influenced by the milk volume taken by the infant (*p* > 0.05). However, when comparisons were made between mother/infant dyads in post-feed samples, the total cell content per mL milk was positively associated with the number of known and total (known + novel) miRNA species (*p* = 0.035 and *p* < 0.001, respectively, *n* = 10), but not with the number of novel miRNAs (*p* = 0.689, *n* = 10). This was in agreement with the associations observed when all pre- and post-feed milk samples from all dyads were considered (see above).

The number of reads was used to identify the most highly expressed miRNAs. The top 19 most highly expressed known miRNAs were consistently present in pre- and post-feed milk, and represented 86.2% of all expressed miRNAs (Figure 3F,G). The top 5 most highly expressed known and top 4 novel miRNAs were validated using qPCR. Expression patterns of the top 5 known miRNAs did not differ between pre- and post-feed milk (*p* > 0.05, *n* = 7) (Figure 2), similar to the sequencing analysis. After normalizing the 5 selected known miRNAs to RNU48, mother with ID 1 showed greater expression of all the 5 miRNAs compared to the other 6 mothers including the reference sample (mother with ID 4). Expression patterns were very similar amongst mothers (Figure 2). LME modeling showed no differences in expression of the 4 novel miRNAs examined between pre- and post-feed milk (*p* > 0.05, *n* = 7) (Figure 2).

**Figure 4:**
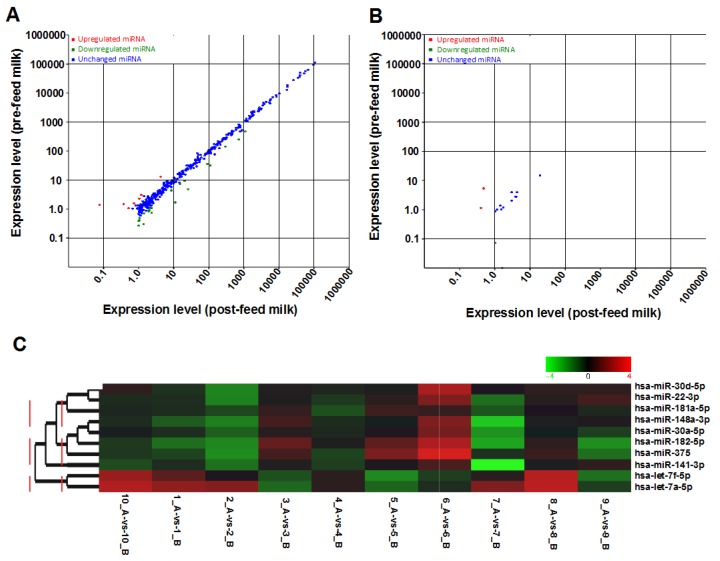
The two differential expression scatter plots show all identified known (**A**) and novel (**B**) miRNAs that were expressed in all pre-and post-feed milk samples (*n* = 10 samples in each group). Each red and green dot represents an individual miRNA that is either up- or down-regulated between pre- and post-feed milk, respectively, whilst blue represents no difference in expression level; (**C**) Heat-map demonstrating the relationship and expression patterns of the top 10 most highly expressed miRNAs between pre-(B) and post-(A) feed milk of individual participants. The expression patterns were analyzed hierarchically by clustering these 10 miRNAs, where green refers to low expression and red refers to high expression level.

Known miRNAs specific to either pre- or post-feed milk were also seen, with 159 miRNAs specific to pre-feed milk that were not identified in any post-feed milk samples, and 180 miRNAs specific to post-feed milk that were not identified in any pre-feed milk samples. These pre- or post-feed milk-specific miRNAs were expressed at low levels (<14 total reads in 10 post-feed milk samples, and <12 total reads in 10 pre-feed milk samples). However, the majority of known miRNAs were identified in both pre- and post-feed milk, with 1128 out of 1467 known mature miRNAs commonly determined in pre- and post-feed milk samples (Table S5). In contrast, most of the novel miRNAs were discovered in either pre- or post-feed milk, and only 180 novel miRNAs were seen in both pre- and post-feed milk. More novel miRNAs were found to be specific to post-feed milk than pre-feed milk samples, with 1035 novel miRNAs only seen in post-feed milk, and 781 novel miRNAs only seen in pre-feed milk (Table S6). The effects of feeding on the expression of known and novel miRNAs in both pre- and post-feed milk samples are shown in scatter plots (Figure 4A,B). Of the miRNAs commonly found in pre- and post-feed milk, 33 known miRNAs were differentially expressed between pre- and post-feed milk (*p* < 0.05, *n* = 10), of which 29 were upregulated in post-feed milk (Table S7). Of the novel miRNAs universally present in both pre- and post-feed milk samples, 3 were differentially expressed between pre- and post-feed milk, of which 2 were upregulated post-feeding (Table S8).

Interestingly, few of the differentially expressed miRNAs were highly expressed, such as hsa-miR-191-5p, which was abundant in both pre- and post-feed milk (total reads = 1,140,649) and was upregulated post-feeding (*p* = 0.002). Most of the top 10 most highly expressed known miRNAs were similarly expressed within a mother/infant dyad (pre- and post-feeding). However in one dyad, hsa-miR-141-3p was downregulated in post-feed milk compared to pre-feed milk, whereas in another dyad hsa-miR-375 was upregulated in post-feed milk. The top 10 most highly expressed miRNAs were clustered together, with a strong correlation in expression in pre- and post-feed milk seen between hsa-miR-30d-5p and hsa-miR-22-3p, and between hsa-let-7f-5p and hsa-let-7a-5p (Figure 4C).

### 2.3. Human Milk Cellular miRNAs Are Regulatory Agents in the Mammary Gland

**Figure 5:**
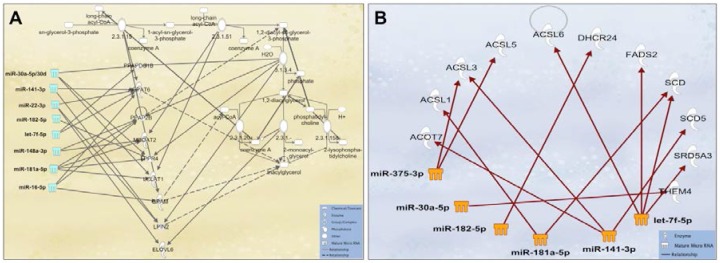
Some of the most highly expressed miRNAs in HM cells are involved in milk synthesis, regulating various pathways within complex molecular networks. (**A**) 8 highly expressed miRNAs (shown in light blue color) were identified to control the production and maintain the level of triacylglycerol in milk fat; (**B**) 6 highly expressed HM cell miRNAs (shown in orange color) that regulated different genes associated with fatty acid biosynthesis, including oleic acid, stearic acid, and palmitic acid.

**Figure 6:**
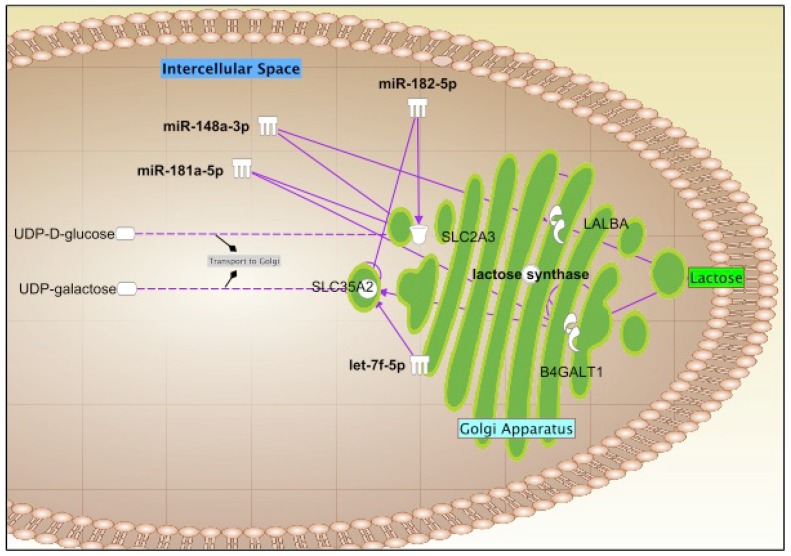
HM lactose synthesis in the mammary gland during lactation is regulated by 4 highly expressed miRNAs in HM (shown in white color). Importantly, miR-182-5p and let-7f-5p regulate SLC2A3 (UDP-glucose transporter) and SL35A2 (UDP-galactose transporter).

Due to the large number of the identified known and novel miRNAs in this study, only the top 10 known and novel miRNAs were used for gene target, GO and KEGG analyses (Table 4). A range of computational approaches was used to predict the target genes of the top 10 miRNAs, including targetscan, RNAhybrid and miRanda. A total of 26,200 targets were predicted for the top 10 known miRNAs (17,586 unique targets), whilst 16,453 targets were predicted for the top 10 novel miRNAs (13,066 unique targets). All identified targets that were regulated by the top 10 most highly expressed known and novel miRNAs were classified using GO and KEGG databases to determine gene functions and metabolic pathways, respectively (Tables S9–S12; Figure S4A,B). Similar to the above target prediction analysis, IPA was used to predict the targets (experimentally confirmed or highly predicted) of the most highly expressed miRNAs (100K reads or above = top 23 miRNAs). 8925 unique targets were determined for the top 23 most highly expressed miRNA (Table S13), with functions in immune response, development, growth, metabolism, and cell cycle (Table 5; Table S14). More specifically, KEGG pathway analysis revealed involvement of the abundant HM miRNAs in many complex metabolic networks, such as glycerophospholipid metabolism, porphyrin and nitrogen metabolism (Tables S11–S12), with the top one targeted pathway being the renin-angiotensin system (RAS) (Figure S5), which controls body fluid balance and blood pressure. Some of the highly expressed and abundant HM cell miRNAs (miR-181a-5p/101-3p/148a-3p/30a-5p/16-5p/141-3p/22-3p/182-5p and let-7f-5p) control ATPase expression and triacylglycerol synthesis (Figure 5A), with the latter forming the basis of HM lipids (Figure 5B), as well as regulate GLUT1 expression, which is associated with lactose synthesis (Figure 6). Furthermore, some of the most highly expressed HM cell miRNAs (miR-181a-5p/375-3p/148a-3p/30a-5p/16-5p/141-3p/22-3p/182-5p/125b-5p and let-7f-5p) control mammary signaling via direct effects on numerous receptors, including the growth hormone receptor (GHR) and its phosphorylation by JAK2 (Figure S6); the insulin-like growth factor-I receptor (IGF-IR); the insulin receptor (INSR) (Figure S7), and estrogen receptor genes (ERα and ERβ) (Figure S8). miRNAs associated with anti-cancer effects in the breast and other organs (miR-181a-5p/148a-3p/30a-5p/141-3p/22-3p/182-5p and let-7f-5p) as well as with immune responses to disease (miR-148a-3p/ miR-181a-5p/182-5p/16-5p/99b/5p and let-7f-5p) were also identified at high expression levels in HM cells.

## 3. Discussion

Human milk is one of the richest sources of miRNAs known to date, with the majority of milk miRNAs being protected within milk cells, fat globules and exosomes, and primarily originating from the lactating epithelium, potentially exerting lactation-specific functions [3,10,11,12,15]. Their high stability in harsh conditions [8,10] and their vehicle-mediated transporting mechanism [3] further increase their likelihood of survival in the gastrointestinal tract of the infant, to subsequently be absorbed into the bloodstream for tissue-specific functions [3]. Indeed, miRNAs derived from bovine milk consumed by humans or animals have been shown to be transferred into the plasma and perform regulatory and therapeutic functions [3,19,23]. HM cells are known to survive the infant’s gut, diapedese through the intestinal mucosa and enter through the bloodstream various tissues, where they integrate and differentiate into functional cells [34]. HM cells are therefore important carriers of miRNAs that provide regulatory signals to the infant. Here, we characterized the miRNA content of HM cells using next generation sequencing, which has only previously been carried out in the lipid and skim milk fractions [10,11]. Numerous known and novel miRNA species were identified, which positively correlated with the number of HM cells (Figure 3C). Although some of the most highly expressed known miRNAs in HM cells were conserved amongst lactating mothers (Figure 3F,G), the variation seen in individual mother-infant dyads (Table S6), particularly in respect to the novel miRNA species, suggests that a component of miRNA-mediated regulation is dyad-specific. Whilst milk removal by the infant does not consistently influence the content and/or expression of miRNAs in HM cells (Figure 4A,B), specific miRNAs increased from pre- to post-feed milk, similar to HM cell and fat content [32].

Similar to previous reports [32], the total cell and fat content of HM increased post-feeding (Figure 3D; Figure S3A), suggesting an association with milk synthesis, cell turnover during breastfeeding, and/or potentially active migration of epithelial cells into the alveolar and ductal lumen [32,35]. When pre- and post-feeding milk samples were considered together (*n* = 20), HM cell content was positively associated with the number of known and total (known + novel) miRNA species, as well as the total miRNA content, and the latter was further related to more known and total miRNA (known + novel) species. However, within mother/infant dyads comparisons examining the effects of feeding on HM cell miRNA (*n* = 10) showed no significant differences in total number of miRNA species and total miRNA content pre- and post-feeding (Figure 3D), something that may be due to the lack of significance in change in HM cell content in this small group (*p* = 0.668 for *n* = 10, whilst *p* = 0.028 in the larger group of *n* = 16 mothers), which is one limitation of the study. Similarly, expression levels of the majority of miRNAs were not different pre- and post-feeding, however a subgroup of 33 known and 3 novel miRNAs were differentially expressed in post-feed milk, of which 29 and 2 were upregulated, respectively (Figure 4A,B). The top 3 included the known hsa-miR-191-5p (total reads 1,140,649) and hsa-miR-30e-3p (total reads 91,722) miRNAs and the novel_mir_39 (total reads 789), which were the most significantly upregulated post-feeding compared to the other differentially expressed known and novel miRNAs (Tables S7 and S8). hsa-miR-191-5p and hsa-miR-30e-3p are known to be involved in cell proliferation and different types of cancer [36]. In particular, hsa-miR-191-5p is upregulated in breast cancer [37,38], and has been suggested as a prognostic marker for breast cancer progression [39]. On the other hand, hsa-miR-30e-3p is a biomarker for inflammatory (autoimmune) disorders [40]. However, in the context of the normal lactating mammary gland, their upregulation post-feeding may be associated with activation of cell division to facilitate the generation of more milk-secretory lactocytes. Functional analysis using GO and KEGG showed that 146 genes involved in fatty acid synthesis are controlled by miRNAs found to be upregulated post-feeding. Moreover, some of these upregulated miRNAs are involved in the production of immunoglobulin A (IgA), which is one of the first lines of defense in the human’s gastrointestinal tract against various infectious diseases [41].

Collectively, these data indicate that towards the end of a feed HM is richer in cells, which in turn contain greater amounts of miRNAs, thus the total cellular miRNA content of HM is likely to be higher in emptier breasts in proportion to the increase in HM cell content with feeding. Considering the potential functional roles of HM miRNA for the infant, feeding on demand likely facilitates exposure of the infant to the full spectrum of HM miRNAs and not only to miRNAs characteristic of milk from fuller breasts. This is further supported by the fact that some miRNAs were detected only in pre- or post-feed milk (Tables S5 and S6) and were therefore specific to either fuller or emptier breasts, suggesting endogenous mammary synthesis of certain miRNAs as the infant removes milk from the breast. These miRNAs may be involved in the regulation of milk synthesis, which is increased as milk is removed from the breast, and/or of the infant’s appetite, and merit further investigation. Indeed, a study has previously shown that increased milk removal from the breast during pumping resulted in upregulation of gene expression of the milk component α-lactalbumin [42], confirming that milk synthesis can be altered by different milk removal regimes.

Munch *et al.* (2013) stated that human milk lipids conserved the highest number of miRNAs (308 mature miRNAs) amongst human milk fractions [11], although skim milk profiled in 2010 using qPCR-based methods showed greater number of miRNAs (429 mature miRNAs) than the Munch *et al.* study on milk lipids [9]. Further, exosomes isolated from skim HM were profiled using Solexa sequencing in 2012, where 602 mature miRNAs were determined [10]. However, the cell fraction of HM was not examined in any of these studies. Here, we provide evidence demonstrating that HM cells are richer in miRNA species than all other human milk fractions, containing a total of 1467 known miRNA species and an additional 1996 novel miRNAs. We used 208,829,362 reads to match the available 2590 known miRNAs to the latest version of miRBase 21.0 (released June 2014), in contrast to Munch *et al.*, who used 124,110,646 reads and an older version of miRBase (14.0), and Zhou *et al.*, who used ~83,520,000 reads matched to miRBase version 17.0. This difference may have also contributed to the higher number of miRNAs identified here. Importantly, 10M reads were used for each individual sample allowing reading of low abundance miRNAs, which as has been previously shown, can be of biological significance [43,44,45]. Moreover, an optimized protocol for HM preparation and miRNA extraction was also used to achieve high efficiency of miRNA isolation [15], in which the analysis of fresh HM and not frozen played an important role.

Target genes were predicted for the top 10 most highly expressed known and novel miRNAs using different computational approaches (Targetscan, RNAhybrid and miRanda). Candidate targets were applied to GO and KEGG to identify their functions in the lactating mammary gland and for the infant. Most of the top 10 known miRNAs are involved in immune responses, development, growth, metabolic processes, reproduction, and exert enzyme regulatory activity. KEGG pathway analysis revealed involvement in many complex metabolic networks, such as glycerophospholipid metabolism, porphyrin metabolism, and nitrogen metabolism. The top one targeted pathway was the renin-angiotensin system (RAS), which is a hormone system controlling body fluid balance and blood pressure. These highly abundant HM miRNAs reaching the infant’s gastrointestinal tract potentially control breastfeeding behavior and appetite via RAS modulation. Further, some of the highly expressed HM cell miRNAs (let-7f-5p, miR-181a-5p/101-3p/148a-3p/30a-5p/16-3p) are known to control ATPase expression including ATP2C1, ATP2B3, ATP2A2, ATP2B4, ATP2B2, and ATP2B1 (Figure S9), and may thus facilitate calcium absorption in the infant since ATPase acts as a ion pump transporting ions including calcium (Ca^2+^) to extracellular space [46]. Indeed, HM calcium is more bioavailable to the infant than that of infant formula [47]. It is of interest that HM contains ample amounts of lactoferrin, which is one of the most active ATPases in HM [48], and may be regulated by these miRNAs.

Notably, highly expressed HM cell miRNAs were significantly associated with disease pathways, such as influenza A and respiratory diseases. These miRNAs are likely to control immunity response to influenza A virus in the infant, enhancing the immunological protection provided via HM. This is in accordance with the numerous previous studies emphasising the protective effects of breastfeeding against infections [49,50,51,52]. For example, exclusive breastfeeding in the first 4-6 months postpartum has been associated with a reduction of upper and lower respiratory infections in infants [53,54]. Further, bioactive components of breastmilk were found to protect against pneumonia, mainly caused by viral infections, during infancy [55,56].

As expected, the miRNA profile of HM cells described in this study reflects the miRNA content and endogenous synthesis in the lactocytes, since that is the dominant cell type in mature HM when both the mother and infant are healthy [4]. In addition to regulatory functions in the infant, highly expressed miRNAs known to regulate triacylglycerol synthesis were identified in HM cells and may be involved in the synthesis of milk lipids in the mammary gland (Figure 5A). Triacylglycerol forms the core of milk fat globules, which mainly contain fatty acids [57,58], and is the basis of human milk lipids (~98%) [59]. HM Fatty acids are derived from *de novo* synthesis in the lactocyte and from blood lipids [60]. Specifically, AGPAT6 (1-acylglycerol-3-phosphate O-acyltransferase 6) is known to be regulated by the some of the top most highly expressed HM cell miRNAs (let-7f-5p, miR-182-5p, miR-148a-3p, and miR-22-3p), and has a direct effect on the synthesis of triacylglycerol and long chain acyl-CoA (fatty acids) [61]. Further to triacylglycerol synthesis, highly expressed HM cell miRNAs are involved in fatty acid biosynthesis including palmitic acid (Figure 5B). FADS2 (fatty acid desaturase 2) that is modulated by let-7f-5p [62], is involved in oleate biosynthesis. Moreover, THEM4 (thioesterase superfamily member 4) is controlled by miR-30a-5p, which is also essential for the phosphorylation and synthesis of fatty acids [62] (Figure 5B).

HM lactose is specifically synthesized in the mammary gland, and is the primary sugar in HM contributing ~40% to the energy intake of the infant [63]. Lactose consists of two different monosaccharides, glucose and galactose that are joined by 1,4 β-glycosidic linkage [60]. In the lactocyte, cytosolic glucose is converted to UDP-galactose by galactose-1-phosphate uridylyltransferase, and is then transported by a glucose transporter (GLUT1) to the lumen of Golgi vesicles [64]. GLUT1 expression is regulated by miR-148a-3p, miR-181a-5p, and miR-182-5p, which were highly expressed in HM cells. UDP-galactose interacts with glucose to synthesize lactose after linking to two different protein complexes in the Golgi membrane, β1-4 galactosyltransferase (β4GalT1) and α-lactalbumin (α-LA) [64]. β4GalT1 is regulated by HM cell miR-181a-5p, whereas α-LA by HM cell miR-148a-3p (Figure 6). Therefore, abundant HM cell miRNA appear to regulate fat and lactose synthesis in the lactocyte, and could potentially be used as indicators of the level of milk synthesis in the mammary gland.Furthermore, some of highly expressed HM cell miRNAs (let-7f-5p, miR-151-3p and miR-16-5p) control the growth hormone receptor (GHR). Human growth hormone (hGH) is critical to milk production in healthy women [65] and variable levels have been reported in human and bovine milk [66]. It is required to maintain lactation performance [60], and has important roles in alveolar and ductal development in the mammary gland [67]. hGH binding to its receptor (GHR) activates Janus Kinase 2 (JAK2), which is mainly responsible for phosphorylation of GH receptor [68]. The HM cell highly expressed miR-375-3p is a regulator of JAK2. GH-activated JAK2 also phosphorylates/activates signal transducers and activators of transcription (STAT) family including STAT1, STAT3 and STAT5 [69]. STATs are involved in many molecular functions including nuclear localization and activation of transcription of target genes [69]. All members of the STAT family are controlled by highly expressed HM cell miRNAs, including miR-181A-5P, miR-30a/d-5p, and miR-141-3p. Suppressor of cytokine signaling (SOCS) protein family is also responsible for termination of GH-activated STAT signaling [68], where the expression of SOCS1-7 proteins is regulated by HM cell miR-182-5p, let-7f-5p, miR-148a-3p, miR-22-3p, miR-16-5p, miR-181a-5p, miR-141-3p (Figure S6). These miRNAs involved in the control of GH-mediated mammary gland development and signaling could potentially be used as indicators of lactation performance.

Importantly, most of the highly expressed HM cell miRNA (let-7f-5p, miR-16-5p, miR141-3p, miR30a/d-5p, miR182-5p, andmiR375-3p) regulate the insulin-like growth factor-I receptor (IGF-IR) (Figure S6). HM contains insulin-like growth factor-I (IGF-I), which is higher in colostrum than mature milk and higher in HM than bovine milk [70]. IGF-I is thought to be efficiently absorbed in the infant’s gastrointestinal tract and to increase in the serum post-feeding [71]. This growth factor is known to be involved in the development and growth of the infant via direct effects on cell differentiation and proliferation [72].

Some of the highly expressed HM cell miRNAs were identified to have critical roles in insulin receptor (INSR) signaling, including miR-181-5p/182-5p/22-3p/141-3p/148a-3p/30a-5p. In addition, INSR itself is regulated by the most highly expressed miRNA in HM, let-7f-5p, and also by miR-182-5p (Figure S7). Insulin via its binding to INSR plays a vital role in regulating different milk components, in particular, glucose and fat homeostasis [73]. Different milk formulae, including standard formula, insulin formula (20 ng/mL) and insulin with trypsin inhibitor (1 U/mL) formula were administrated to rat pups, resulting in increase in plasma insulin, with a direct positive effect on pancreatic amylase activity required in early life [74]. Further, Insulin receptor (INSR) was detected in the epithelial cell of piglet intestine, suggesting that milk insulin plays a crucial role in the development of the newborn intestine, passing from the GI tract to the bloodstream [75]. Indeed, insulin in HM is up to 30-fold higher than in infant formula [76]. INSR expression increases in response to nutrients to reduce glucose and synthesis of glycogen [73]. Controlling the INSR via miRNAs in blood has been previously investigated, where some miRNAs were shown to inhibit INSR [77]. Human milk miRNAs potentially control the signaling of insulin receptor based on the needs to decrease glucose.

Highly expressed miRNAs in HM cells are also associated with regulation of estrogen receptor genes (ERα and ERβ) (Figure S8), which may play important functions in maintaining a normal milk supply. High levels of estrogen inhibits milk production, thus it is present in very low levels to allow prolactin (PRL) to maintain milk synthesis [78]. It has been reported that miR-21, miR-125b and miR-143, which are highly expressed in HM, positively modulate PRL receptor in dairy cow mammary gland epithelial cells (DCMECs) to activate of STAT5 [79]. Further, the abundant in HM cells miR-181A-5P, miR-22-3p and miR-21-5p repress ERα (ESR1), whilst the milk most highly expressed miRNA, let-7f-5p, negatively regulates ERβ (ESR2) (Figure S8). Estrogen receptors (ERs) are overexpressed in ER+ breast cancer, which is the most common type of breast cancer [80]. The known treatment of this cancer is based on blocking the estrogen hormone to bind the excessive ERs on cancer cells, preventing cancer cell proliferation [80]. Highly expressed HM miRNAs synthesized in the mammary gland, which target ERs, could be used as a novel therapeutic approach in this cancer [81].

Given the high metabolic rate of the lactating mammary gland, it was not surprising that miRNAs known to act as oncogenes, such as miR-21-5p [82], were found to be highly expressed in HM. In the context of lactation, these HM miRNAs are likely to play important functions in the normal remodeling of the lactating mammary gland, and/or the development of the breastfed infant. They may also be useful biomarkers of lactation performance and the health status of the mammary gland [83], and require further investigation.

In summary, our study demonstrates that HM cells are rich in miRNA species and content compared to other HM fractions, such as lipids and skim milk, and all other human body fluids. Robust miRNA prediction analysis revealed numerous novel miRNAs, some of which may be specific to lactation. Milk removal by the infant during breastfeeding influences a small subset of HM miRNA species and their expression levels, and results in richer miRNA content in post-feed milk due to the higher number of cells and their associated miRNAs. The identified miRNA targets reveal critical potential roles of miRNAs in the infant and the lactating gland, and support their use as diagnostic biomarkers for both lactation performance and breast health.

## 4. Materials and Methods

### 4.1. Ethics Statement and Sample Collection

This study was approved by the Human Research Ethics Committee of The University of Western Australia, and all methods were conducted in accordance with the approved guidelines (Figure S10). All participants provided informed written consent. Exclusively breastfeeding mothers in month 2 of lactation (weeks 4 to 8) were recruited in this study (*n* = 16), and were all healthy, including their infants, at the time of sample collection. A volume of 5 mL of milk was obtained before a morning breastfeeding session (pre-feed sample) from the fuller breast, and then a second 5-mL milk sample was collected from the same breast immediately after feeding (post-feed sample). The feeding sessions lasted for at least 5 min and the amount of milk taken by the infant was measured by weighing the infant before and after feeding, as described previously [32]. Samples were collected aseptically using an electric breast pump (Medela AG, Baar, Switzerland), and were immediately transported to the laboratory for milk fractionation and miRNA extraction.

### 4.2. Cell Isolation and miRNA Extraction from Human Milk

HM fat content was measured as previously described [12]. HM was fractionated into cells, skim milk and lipids as described previously [12,84]. Briefly, freshly expressed HM was diluted 1:1 with PBS (Gibco, Thermo Fisher Scientific, Waltham, MA, USA) and centrifuged at 800× *g* for 20 min at 20 °C. Purified milk cells were washed in PBS at 800× *g* for 5 min at 20 °C, and were then counted using a haemocytometer as previously described [84]. miRNA were extracted from all samples immediately without cryopreservation using the miRNeasy mini Kit (Qiagen, Hilden, Germany) according to previous optimization studies [15]. The concentration and purity of miRNAs were measured using a NanoDrop 2000 Spectrophotometer (Thermo Scientific, Wilmington, MA, USA) and an Agilent Bioanalyzer 2100 instrument (Agilent, Santa Carla, CA, USA) with the RNA 6000 Nano Chip kit (Agilent, Santa Carla, CA, USA). All extracted miRNA samples were stored at −80 °C, and pre- and post-feed samples from a subgroup of 10 mothers were used for small RNA sequencing and qPCR validation (Figure S10).

### 4.3. Statistical Analysis of Human Milk Composition

All analyses were performed using R Studio Version 0.98.1103 package [85]. The additional packages nlme [86] and lattice [87] were used for linear mixed effects modeling (LME) and graphical exploration of the data, respectively. Differences were considered to be significant if *p* < 0.05. General linear hypothesis tests and LME were used to determine the differences between pre- and post-feed samples in total cell content, fat content, miRNA content and species number, as well as account for the effect of milk intake and other demographic characteristics of individual mother/infant dyads.

### 4.4. Library Construction, Small RNA Sequencing, and Bioinformatics Analysis

All human milk cell miRNA samples to be analyzed using small RNA sequencing (*n* = 20) were standardized to 500 ng/μL. Libraries were created for each sample individually as previously described [88,89]. Briefly, by using size fractionation, 18–30 nt in length of small RNAs (sRNAs) were obtained and ligated to 5′-RNA and 3′-RNA adapters. cDNA was created using small RNA primers. All cDNA were sequenced into two SE50 lanes, where ~10M reads were generated for each sample using Illumina HiSeq 2000 platform (Figure S10). Cleanup reads were done on raw reads to trim low or contaminate reads such as 5′ primer contaminants, no insert tags, oversized insertion, low quality reads, poly A reads, *etc.* Clean reads were distributed by length, where sRNAs were considered between 18–30 nucleotides in length. This step was utilized to identify miRNAs within different sRNAs (for example, miRNA is usually between 21 and 22 nt in length, siRNA is 24 nt, and piRNA is 30 nt). Further, the clean RNA reads were annotated by BLAST into different categories against Rfam (ftp://sanger.ac.uk/pub/databases/Rfam/) and GenBank (http://blast.ncbi.nlm.nih.gov/) to determine miRNA, siRNA, piRNA, rRNA, tRNA, snRNA, snoRNA, and repeat associated sRNA. Degraded fragments of mRNA within sRNAs were detected by alignment to exons and introns of mRNAs. Reads were then mapped to the human genome by bowtie to analyze their expression and distribution on the human genome. Any mapped sRNAs to exons, introns or intergenic regions of the human genome, which did not match any other RNAs, were predicted as novel miRNAs. Further, miRNAs were mapped to miRBase 21.0 (released June 2014) (http://www.mirbase.org/) using BLAST to identify human known mature miRNAs and their precursors. Unmatched miRNAs to known mature miRNAs in miRBase were mapped again to the human genome using the SOAP software to predict potential miRNAs. Usually, the characteristic precursor structure of miRNAs (pre-miRNAs) must be designed to predict potential novel miRNAs. The Mireap software (http://sourceforge.net/projects/mireap/) was used to predict the stem loop (pre-miRNAs) for novel mature miRNAs by exploring the secondary structure, the dicer cleavage site, and the minimum free energy of the unannotated small RNA reads. Then, potential miRNAs were further assessed by identification of base bias on the first position and the nucleotide length on each position.

### 4.5. Differential Expression Analysis

A comparison of the expression of both known and novel mature miRNAs between pre- and post-feed milk samples was done to investigate differentially expressed miRNAs. Expression between pre- and post-feed milk samples was normalized to obtain expression levels of transcript per million (TPM) using the following normalization formula: normalized expression = actual miRNA count/total count of clean reads × 1,000,000. Fold change was calculated using the following formula: Fold change = log_2_(normalized expressed miRNA from post-feed milk/normalized expressed miRNAs from pre-feed milk). DEGseq (R package) [90] was used to determine *p*-values for fold expression change between pre- and post-feed samples, and to generate scatter plots. miRNA with *p* < 0.05 was considered to be differentially expressed miRNA between pre- and post-feed milk.

### 4.6. qPCR Validation

The top 5 most highly expressed known miRNAs and the top 4 novel miRNAs across all samples tested were used for qPCR validation of their presence and expression levels in pre- and post-feed HM samples from *n* = 7 mothers. Milk samples were standardized equally to 500 ng of total miRNA. The known miRNAs examined were: hsa-let-7f-5p, hsa-miR-181a-5p, hsa-miR-148a-3p, hsa-miR-22-3p, and hsa-miR-182-5p. The novel miRNA sequences examined are listed below, with all primers and probes synthesized by Life Technologies and assayed using custom TaqMan small RNA (Thermo Fisher Scientific, Waltham, MA, USA): novel_mir_7-p5 UCCAUAUCCCAACCUGUCAGAGU, novel_mir_299-5p ACUAGGAUUGUGCUUCCCUGG, novel_mir_367-3p UGCACGCGACCAUAGAGCCU, novel_mir_39-5p UCUGGCAUGGCCUUGGGCACU. Reverse transcription and qPCR reaction were performed as previously described [15]. Relative quantitation (RQ) was obtained using 7500 software V2.0.6, and was compared between samples in the R Studio Version 0.98.1103 package [85] using linear mixed effects (LME) models, where *p* ≤ 0.05 was considered statistically significant.

### 4.7. Target Prediction and Functional Analysis

Three different databases/algorithms were used to predict gene targets of all identified miRNAs and of the top 10 most highly expressed known and novel miRNAs. These are targetscan (http://www.targetscan.org/), RNAhybrid (http://bibiserv.techfak.uni-bielefeld.de/rnahybrid), and miRanda (http://www.microrna.org/microrna/home.do). Determined target genes (identified in two or three of the above databases) were used for the functional analysis, where the predicted target gene candidates of known and novel miRNAs were annotated to predict the number of genes involved in different cellular and signaling functions using the Gene Ontology (GO) database (http://www.geneontology.org/) [91]. These target genes were classified into three enriched GO terms (cellular component, molecular function and biological process). This was conducted by mapping target gene candidates to GO terms using its database (http://www.geneontology.org/). The hypergeometric test was used to predict the significant GO terms in target gene candidates. Furthermore, enriched metabolic pathways were determined for target gene candidates using Kyoto Encyclopaedia of Genes and Genomes (KEGG) (http://www.genome.jp/kegg/) [92].

### 4.8. Analysis of Pathways, Networks, and miRNA Gene Targets

The top 700 most highly expressed known mature miRNAs in HM cells (total count ≥ 40 reads) in all sequenced samples (*n* = 20) were uploaded and analyzed in QIAGEN’s Ingenuity Pathway Analysis (IPA^®^, QIAGEN, Redwood City, CA, USA www.qiagen.com/ingenuity). This computer simulation software was used to determine the possible interactions and relationships of the identified highly expressed known miRNAs in different signaling and metabolic pathways. Further, it was used to build molecular networks between the target genes and the identified highly expressed mature known miRNAs through the 6–8 seed region between mRNA and miRNA. Target genes of the uploaded miRNAs (100K reads or above = top 23 miRNAs) were identified using different databases including TarBase, miRecords, Targetscan (IPA tool “MicroRNA Target Filter”), and the IPA findings. All identified target genes were either experimentally verified or were strongly predicted to interact with mature miRNA sequences. Functional analyses included biological roles and related diseases that were associated with more miRNA species (100K reads or above = top 23 miRNAs). *p*-value was calculated using Fisher’s exact test to determine pathways (biological function and/or related disease) that significantly associate with the uploaded unique miRNAs.

### 4.9. Availability of Supporting Data

All raw small RNA sequences are available in the NCBI Gene Expression Omnibus database (Bethesda, MD, USA) under accession number GSE71098. Additional information is also included as supplementary files.

## Figures and Tables

**Table 1:** A list of all known and novel miRNA species number in each sample with the total number of reads that were generated from small RNA sequencing using Solexa.

Sample ID *	Number of Known miRNAs	Number of Known miRNA Reads	Number of Novel miRNAs	Number of Novel miRNA Reads
1_B	790	10,037,707	107	1507
1_A	859	9,203,046	74	1374
2_B	563	6,483,425	209	2267
2_A	532	8,344,307	249	3667
3_B	840	10,137,502	108	1182
3_A	876	9,424,413	103	1045
4_B	820	9,539,244	90	1138
4_A	814	9,694,427	90	1242
5_B	750	5,658,703	35	366
5_A	823	8,164,895	53	709
6_B	833	9,378,155	140	1410
6_A	899	8,811,523	131	1224
7_B	468	6,534,047	184	1918
7_A	662	8,058,217	157	1268
8_B	759	9,732,524	115	1240
8_A	707	7,599,182	118	1178
9_B	750	7,744,119	143	1310
9_A	586	7,897,409	222	2009
10_B	789	10,327,701	179	1864
10_A	780	11,415,988	330	3405
	Total: 1467	174,186,534	Total: 1996	31,323

* B refers to milk before feed (pre-feed milk); A refers to milk after feed (post-feed milk).

**Table 2:** Number of known and novel miRNAs in each sample group (pre- and post-feed milk) with the total reads (expression level).

Sample Group	Pre-Feed Milk (*n* = 10)	Post-Feed Milk (*n* = 10)
Number of known miRNAs	1287	1308
Total reads (known)	85,573,127	88,613,407
Number of novel miRNAs	961 (35 *)	1215 (41 *)
Total reads (novel)	14,202 (5282 *)	17,121 (6950 *)
Number of specific miRNAs	159	180

* High confidence novel miRNAs: novel miRNAs with > 20 total reads and identified in ≥ 4 samples.

**Table 3:** The mean ± standard deviation of HM cell content, and miRNA content and quality using NanoDrop 2000 and Bioanalyzer 2100 respectively, in pre-feed and post-feed milk samples (*n* = 10 each).

	Mean ± S.D. of the Number of Total Milk cells/mL Milk (Cell Viability %)	Mean ± S.D. of Total miRNA Content (ng/10^6^ cells)		Mean ± S.D. of miRNA Quality (OD 260/280) & RIN	
All samples (*n* = 20)	1,222,860 ± 767,091 (92.7%)	NanoDrop	1414 ± 519	NanoDrop	2.05 ± 0.05
		Bioanalyzer	1000 ± 438	Bioanalyzer	8.67 ± 1.22
Pre-feed milk (*n* = 10)	1,146,364 ± 843,594 (91.3%)	NanoDrop	1391 ± 571	NanoDrop	2.04 ± 0.06
		Bioanalyzer	996 ± 481	Bioanalyzer	8.57 ± 1.69
Post-feed milk (*n* = 10)	1,299,356 ± 719,432 (93.3%)	NanoDrop	1438 ± 490	NanoDrop	2.05 ± 0.04
		Bioanalyzer	1004 ± 417	Bioanalyzer	8.77 ± 0.52

**Table 4:** Top 20 most highly expressed known and novel miRNAs across all 20 pre- and post-feed milk samples with the total reads, and the number of samples that each miRNA was detected in.

	Known miRNA	Number of Reads	Number of Samples Detected in (*n* = 20)	Novel miRNA	Number of Reads	Number of Samples Detected in (*n* = 20)
1	hsa-let-7f-5p	25,479,884	20	novel_mir_7	3890	13
2	hsa-miR-181a-5p	21,601,482	20	novel_mir_299	942	19
3	hsa-miR-148a-3p	14,925,495	20	novel_mir_367	804	17
4	hsa-miR-22-3p	12,999,002	20	novel_mir_39	789	20
5	hsa-miR-182-5p	11,288,364	20	novel_mir_115	760	17
6	hsa-let-7a-5p	10,343,580	20	novel_mir_476	658	4
7	hsa-miR-375	9,145,308	20	novel_mir_90	586	17
8	hsa-miR-141-3p	8,577,962	20	novel_mir_41	341	12
9	hsa-miR-30a-5p	7,844,936	20	novel_mir_269	322	15
10	hsa-miR-30d-5p	6,173,556	20	novel_mir_161	295	9
11	hsa-miR-146b-5p	4,140,259	20	novel_mir_278	247	14
12	hsa-miR-26a-5p	3,876,162	20	novel_mir_76	222	13
13	hsa-miR-21-5p	3,457,523	20	novel_mir_430	194	14
14	hsa-let-7i-5p	2,264,279	20	novel_mir_144	189	3
15	hsa-miR-92a-3p	1,918,918	20	novel_mir_456	178	9
16	hsa-miR-27b-3p	1,849,454	20	novel_mir_202	174	12
17	hsa-miR-181b-5p	1,594,211	20	novel_mir_159	173	12
18	hsa-miR-423-5p	1,374,443	20	novel_mir_411	169	11
19	hsa-miR-125a-5p	1,260,164	20	novel_mir_251	156	8
20	hsa-miR-10a-5p	1,145,612	20	novel_mir_425	154	7

**Table 5:** Number of highly expressed experimentally validated known miRNAs (reads > 100K) involved in different molecular, cellular and developmental functions, or disease.

Top Function or Disease Involvement	*p* Value	Number of miRNAs
Molecular and Cellular Functions		
Cell development	5.00 × 10^−2^–6.09 × 10^−10^	24
Cell growth and proliferation	4.88 × 10^−2^–6.09 × 10^−10^	26
Cell movement	4.21 × 10^−2^–1.17 × 10^−6^	15
Cell death and survival	4.41 × 10^−2^–2.45 × 10^−5^	17
Cell cycle	3.82 × 10^−2^–3.80 × 10^−5^	9
Physiological System Development and Function		
Organismal development	4.39 × 10^−6^–9.21 × 10^−10^	5
Digestive system development and function	2.54 × 10^−6^–2.54 × 10^−6^	4
Hepatic system development and function	2.54 × 10^−6^–2.54 × 10^−6^	4
Organ development	1.63 × 10^−2^–2.54 × 10^−6^	5
Connective tissue development and function	6.13 × 10^−3^–3.80 × 10^−5^	4
Diseases and Disorders		
Cancer	4.41 × 10^−2^–1.21 × 10^−34^	41
Hematological diseases	3.01 × 10^−2^–1.21 × 10^−34^	26
Immunological diseases	3.01 × 10^−2^–1.21 × 10^−34^	24
Organismal injury and abnormalities	4.41 × 10^−2^–1.21 × 10^−34^	41
Reproductive system diseases	2.60 × 10^−2^–2.10 × 10^−28^	31

## Supplementary Materials

Supplementary materials can be found at http://www.mdpi.com/1422-0067/17/6/956/s1.
