# Supplementary material for: Human Milk Cells Contain Numerous miRNAs that May Change with Milk Removal and Regulate Multiple Physiological Processes
Source: Int J Mol Sci. 2016 Jun 17;17(6):956. doi: 10.3390/ijms17060956 (PMC4926489; doi:10.3390/ijms17060956)
Supplement: Supplementary file 1 [file ijms-17-00956-s001.zip › ijms-130614-Supplementary Materials/ijms-130614-suppl. figures.pdf]

Supplementary Materials: Human Milk Cells Contain Numerous miRNAs that May Change with Milk Removal and Regulate Multiple Physiological Processes

Mohammed Alsaweed, Ching Tat Lai, Peter E. Hartmann, Donna T. Geddes and Foteini Kakulas

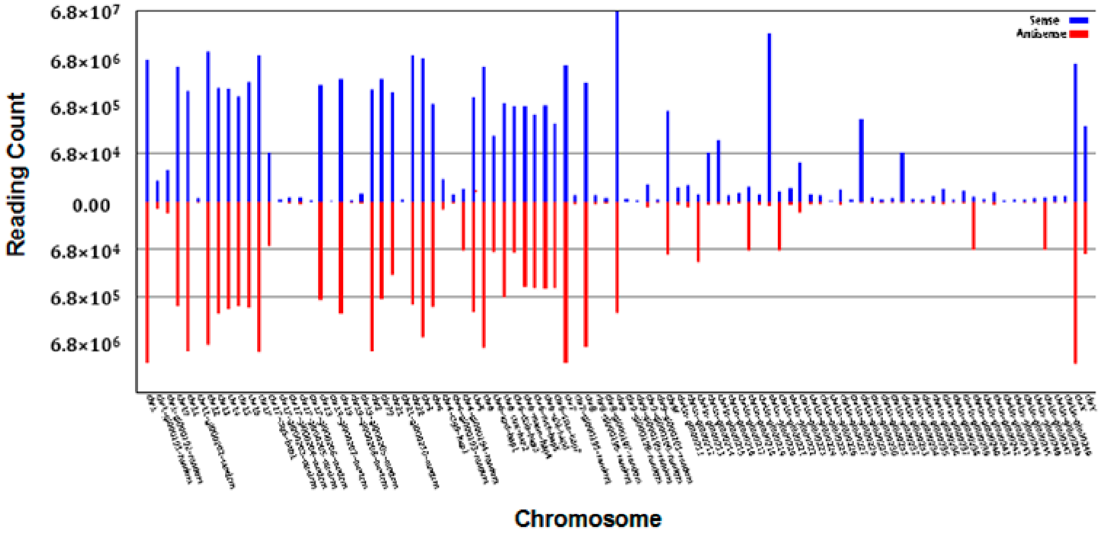

Figure S1. The distribution and number of clean reads that mapped to the human genome. Red bars refer to the sRNAnumber on the antisense strand of the chromosome, whilst blue bars refer to the sRNA number on the sense strand of the chromosome.

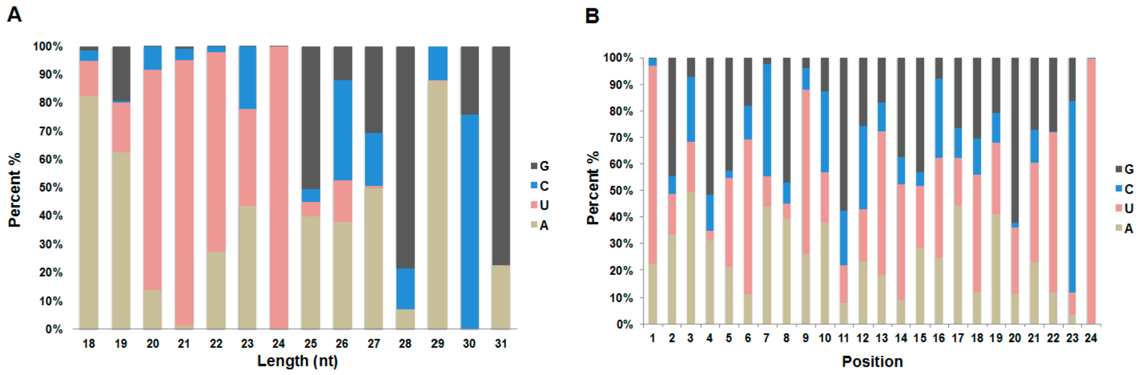

Figure S2. (**A**) The first nucleotide bias from the 5' end of sRNA tags between 18 and 31. Each color represents the four bases, with the y axis indicating the percentage of each base; (**B**) Base bias of all identified known miRNAs at each nucleotide position, with y axis indicating the percentage of each base.

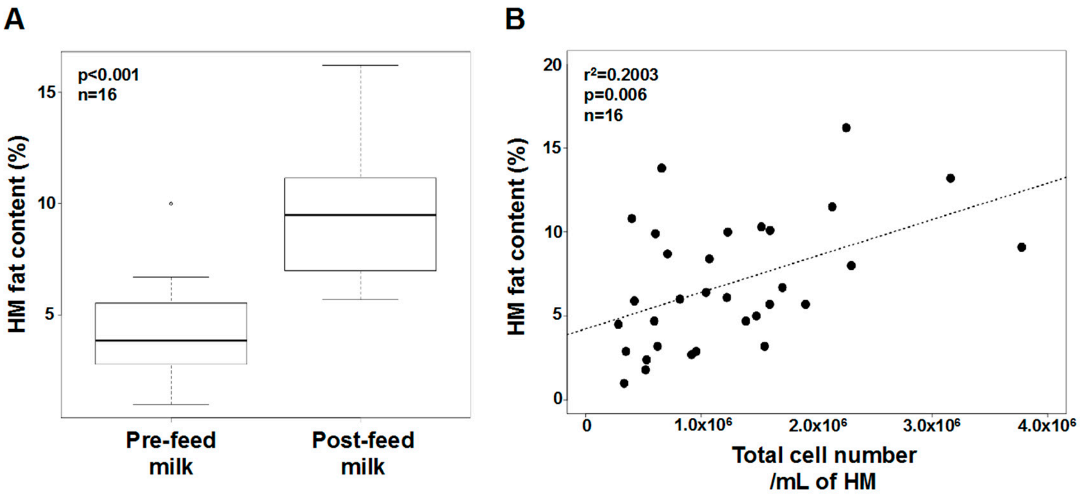

Figure S3. (**A**) Boxplots showing the percentage of human milk fat content in pre- and post-feed milk samples; (**B**) Association between fat and cell content of human milk samples examined.

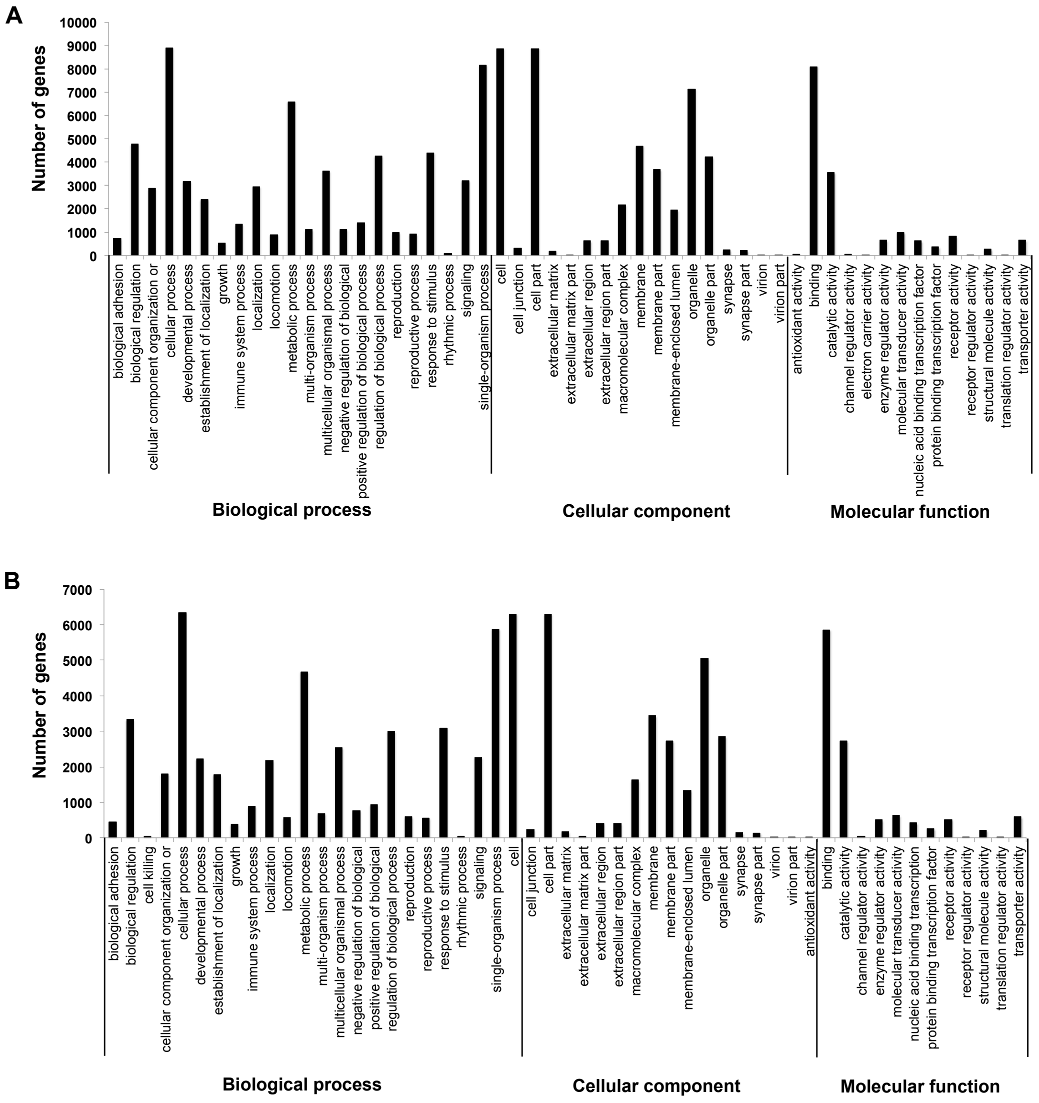

Figure S4. Enriched targets of (**A**) the top 10 known miRNAs; and (**B**) the top 10 novel miRNAs, annotated to different functions on the Gene Ontology (GO).

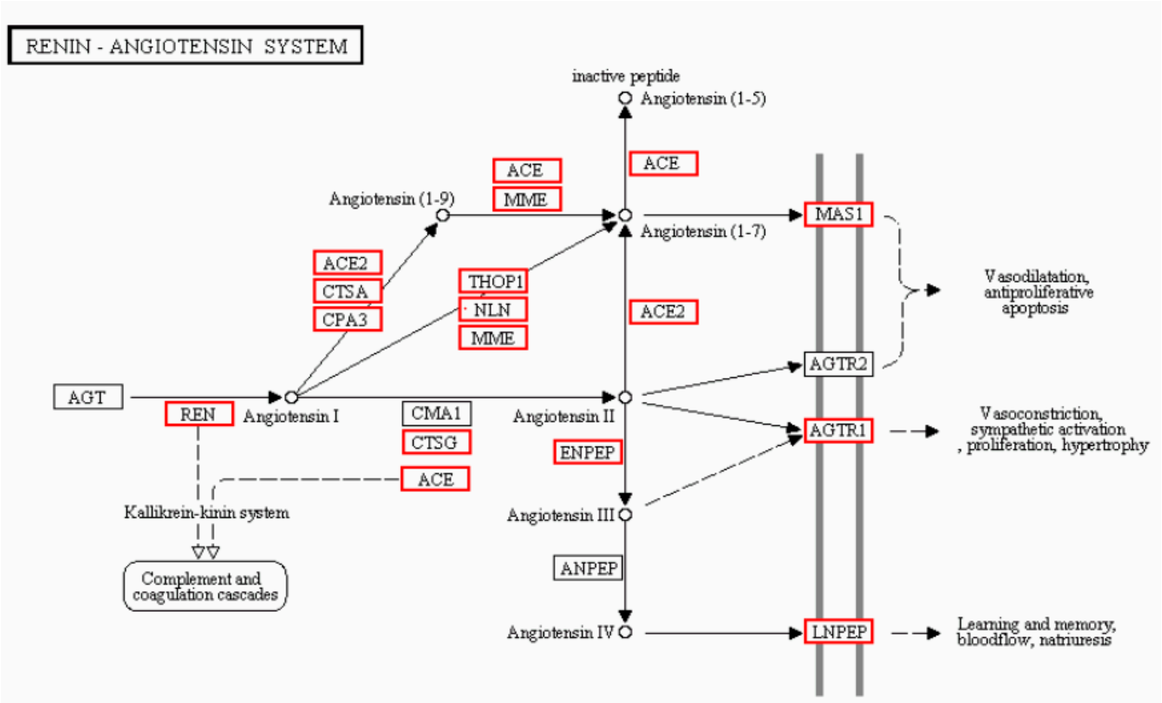

Figure S5. Target genes regulated by the top 10 most highly expressed known miRNAs that encode proteins in the KEGG renin- angiotensin system (RAS). Most of the upregulated genes in the RAS pathway (red squares) are controlled by the top 10 human milk cell miRNAs.

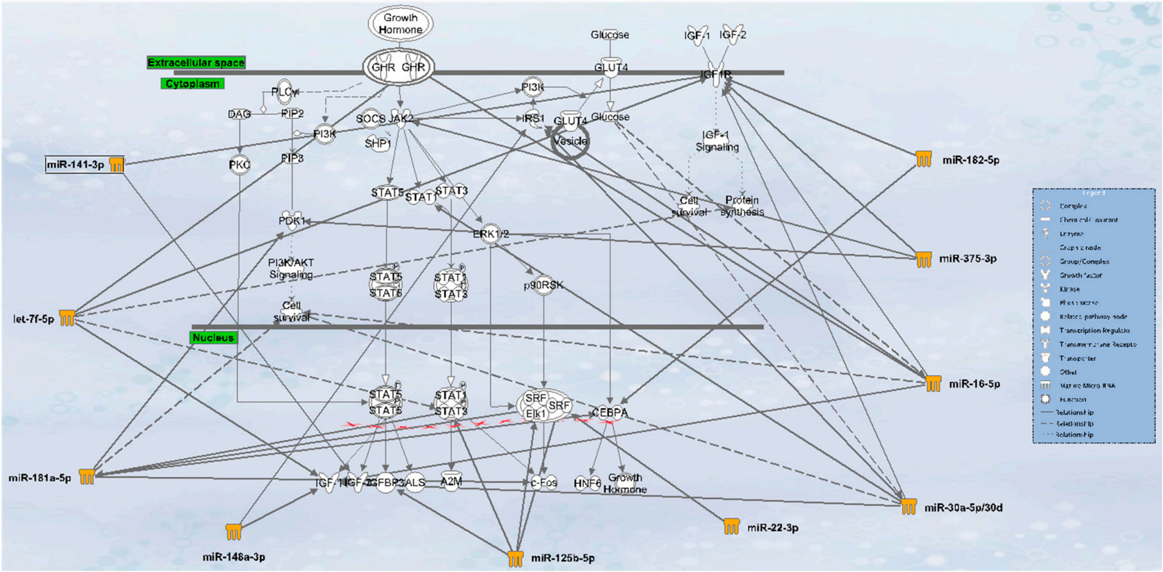

Figure S6. Amongst the top 20 most highly expressed miRNAs in human milk, 10 miRNAs were identified to regulate the synthesis of growth hormone and growth hormone receptors Phosphorylation of GH receptor through JAK2 is controlled by miR-375-3p. Also, the STAT gene family is controlled by some of the most highly expressed human milk miRNAs, including miR-181A-5P, miR-30a/d-5p, and miR-141-3p.

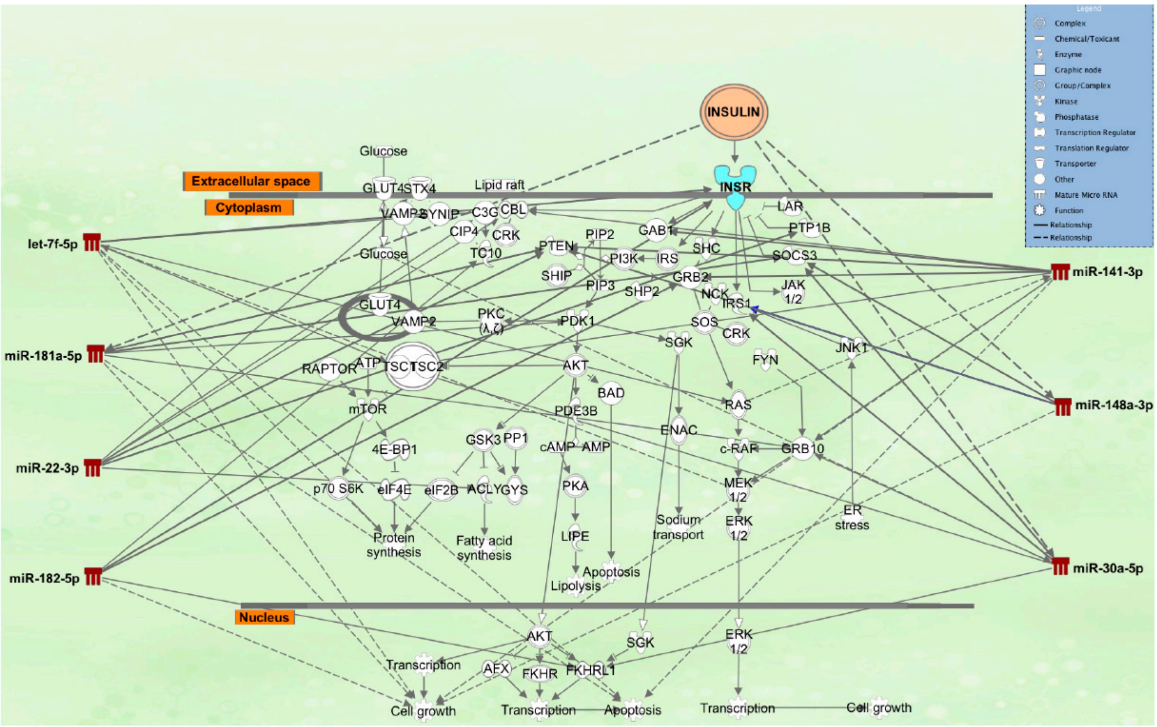

Figure S7. miRNAs enriched in human milk (miR-181-5p/182-5p/22-3p/141-3p/148a-3p/30a-5p) enhance insulin production and regulate the insulin receptor (INSR).

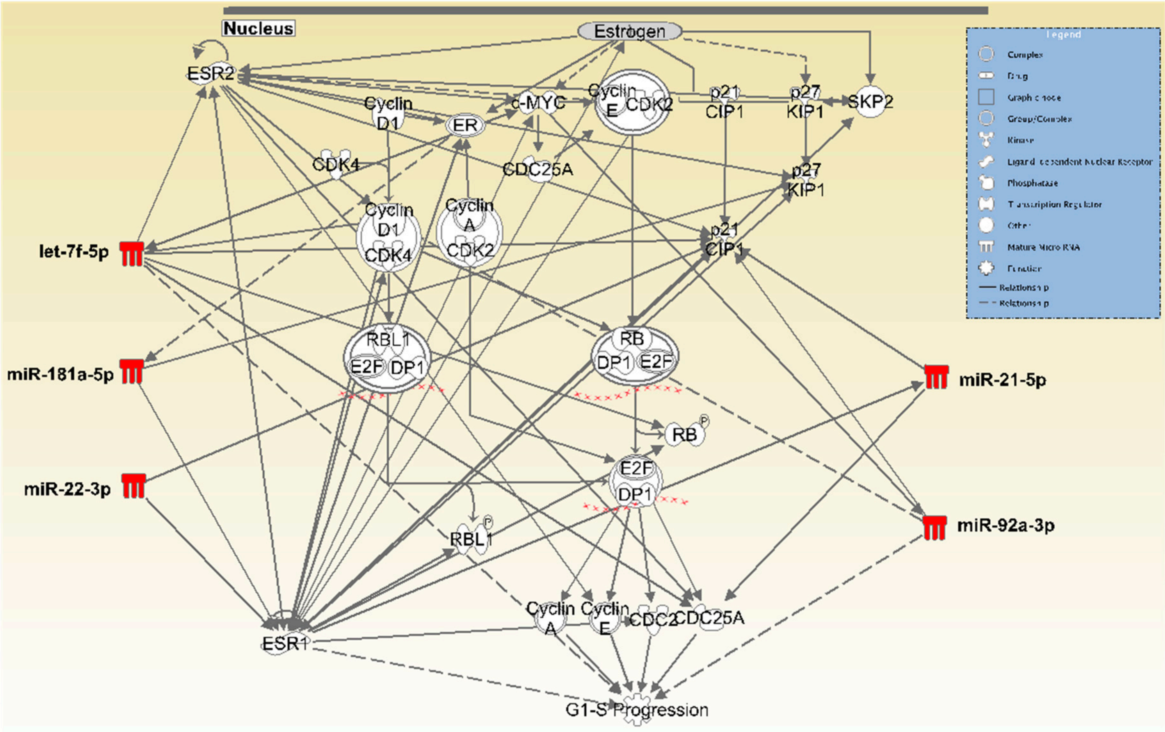

Figure S8. Five of the top 10 most highly expressed miRNAs in human milk modulate estrogen production and the receptors ESR1 and ESR2. Specifically, miR-181A-5P, miR-22-3p and miR-21-5p were identified to control ERα (ESR1), whilst the milk highest expressed miRNA, let-7f-5p, negatively controls ERβ (ESR2).

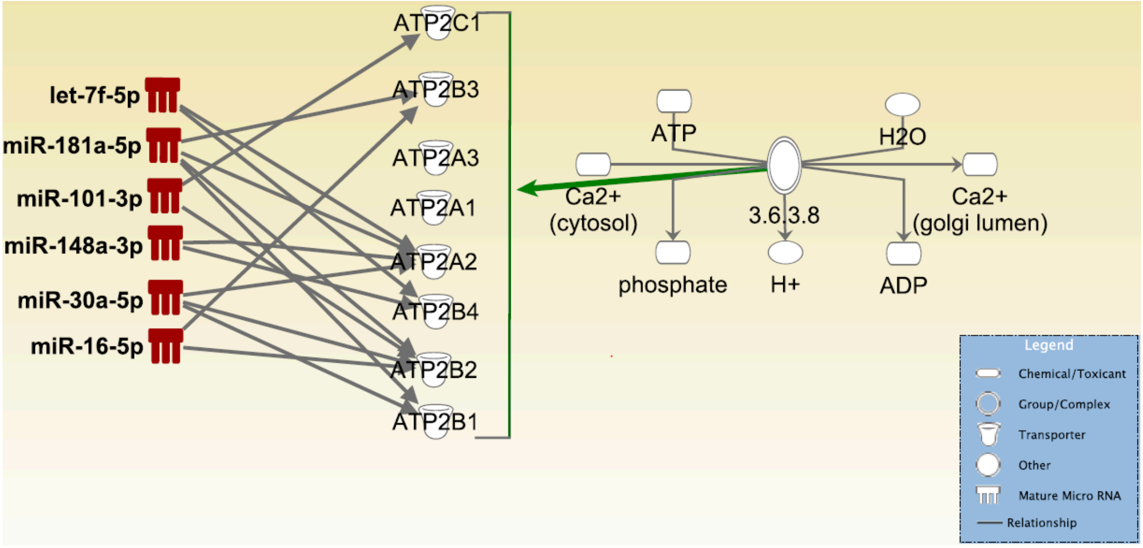

Figure S9. Calcium transport (calcium pump) through different ATPases (ATP2C1, ATP2B3, ATP2A2, ATP2B4, ATP2B2, and ATP2B1) in the mammary gland is modulated by a group of highly expressed miRNAs in human milk (let-7f-5p, miR-181a-5p/101-3p/148a-3p/30a- 5p/16-3p).

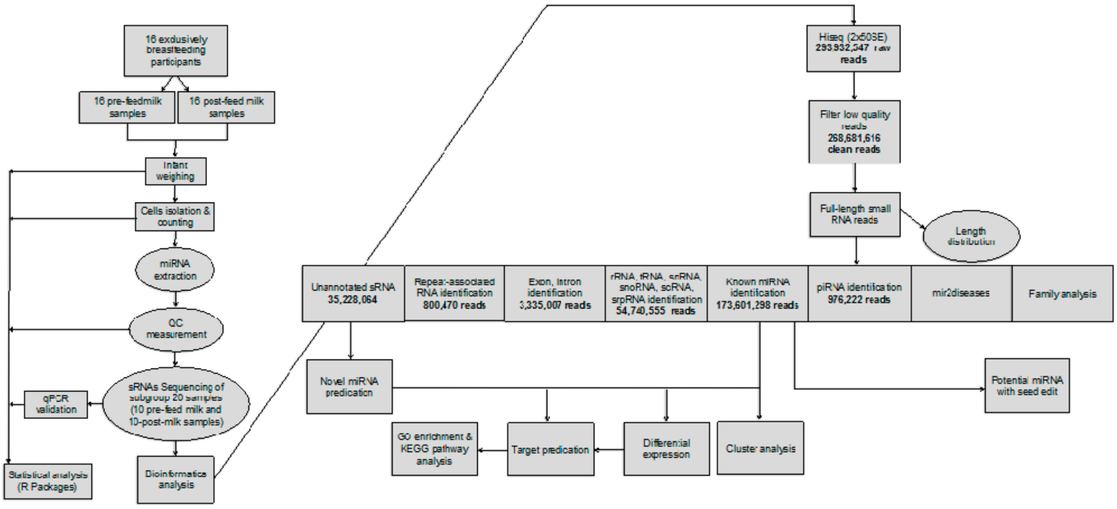

Figure S10. Flowchart describing the study. (A) 16 exclusively breastfeeding mothers were recruited and cells were isolated from individual samples. Small RNAs were sequenced using Solexa in a subgroup of 10 mothers (20 samples, 10 pre- and 10 post-feed milk); (B) Bioinformatics analysis was conducted to determine known miRNAs and predict novel miRNAs, and also to identify their targets and functions.
